# Medicinal Plants and Natural Active Compounds for Cancer Chemoprevention/Chemotherapy

**DOI:** 10.1155/2017/7952417

**Published:** 2017-04-09

**Authors:** Hilal Zaid, Michael Silbermann, Alaa Amash, Dan Gincel, Essam Abdel-Sattar, Nazli B. Sarikahya

**Affiliations:** ^1^Qasemi Research Center, Al-Qasemi Academic College, P.O. Box 124, 30100 Baqa Al-Gharbiyye, Israel; ^2^Faculty of Arts and Sciences, Arab American University of Jenin, P.O. Box 240, Jenin, State of Palestine; ^3^Technion-Israel Institute of Technology, Middle East Cancer Consortium, Haifa, Israel; ^4^The Research Institute of the McGill University Health Centre, Montreal, QC, Canada; ^5^Maryland Technology Development Corporation (TEDCO), Columbia, MD, USA; ^6^Department of Pharmacognosy, College of Pharmacy, Cairo University, Cairo, Egypt; ^7^Department of Chemistry, Faculty of Science, Ege University, Bornova, 35100 Izmir, Turkey

Cancer is a cohort of diseases in which abnormal cells divide without control and are able to invade other tissues (through the blood and lymph systems). Cancer has devastating consequences for the patients' life and indeed is a leading cause of death worldwide. More than 100 different types of cancer are known and are usually named by the organ or type of cell where they start; for example, a cancer that begins in the colon is called colon cancer. The growing incidence of these diseases due to the rising age of the population poses a considerable burden on the public health system. Statistical studies indicate that cancer strikes more than one-third of the population and it is the cause of more than 20% of all deaths. Cancer is caused usually due to abnormalities in the DNA of the affected cells leading to an extra mass of tissue called a tumor. Tumors may be benign (not cancer) or malignant (cancer) [1, 2].

The incidence of cancer might increase in many tissues because of chronic inflammation (e.g., all gastrointestinal). Cancer may develop in cells that are selected for resistance to inflammatory products by virtue of overexpressing antiapoptotic proteins [3]. Current treatments for cancer, besides surgery, are heavily based on cytotoxic regimens of compounds and radiation that interfere with the cellular replication system and thus aim to primarily target rapidly dividing cells. However, due to low selectivity, these treatments show many side effects and are, more importantly, becoming ineffective due to the development of resistance. A promising and new approach to fight cancer is to develop agents that abrogate the cancer's capability to become resistant. Herbal derived drugs (e.g., polyphenols, brassinosteroids, and taxol) are desired for anticancer treatment, as they are natural and readily available. Currently, a few plant products are being used to treat cancer. However, the molecular mechanism of the anticancer effect of some of those plants products and dissecting new products from those plants await further studies [2].

This special issue provides a comprehensive overview on anticancer traditional herbal medicine. The greater majority of contributions arrived from the Far East (China, Korea, Japan, and Taiwan) and the rest from Africa (Uganda, Algeria) and the Middle East (Palestine). We were highly impressed by both the design of the experiments and their high-class execution. The technologies used are up-to-date and convincing. Most of the studies in this issue applied in vitro (using established cell line) and in vivo (animal models and human patients) tests to evaluate the efficacy of anticancer medicinal plants and phytochemicals activity and their action of mechanism. The current special issue involves a relatively large variety of cancers such as leiomyoma, melanoma, hepatoma, breast, prostate, gastric, thyroid, colorectal, osteosarcoma, and squamous cell cancer, while addressing features such as apoptosis and growth factors as the indicative factors.

By and large, this new special issue contributes a significant and substantial collection of well documented data to our armamentarium in fighting cancer. An additional issue to be emphasized is the fact that the herbal remedies are not confined to the effort to cure cancer but also to alleviate cancer patients undergoing treatment: chemotherapy. We are convinced that, by increasing the quality of life especially during the last stage of the cancer journey, we are doing an enormous service to the suffering patient and his caring relatives. As physicians, we should never forget that, concomitant with trying to cure the tumor, our obligation is to care for the patient as a human being keeping his self-dignity until his last day and hours. The nonconventional approach of using natural herbal substances provides us with an important tool to minimize pain, suffering, and hopelessness. It is for that reason that we encourage more scientists and clinicians to proceed with their scientific work of characterizing more natural compounds and try to adjust them for clinical use.

Complementary medicine has suffered for many years from lack of solid evidence in order to justify its use in clinical medicine. It appears that we have reached the phase that this claim is less and less valid, as more evidence-based products are allowed to be commercialized and used under the supervision of an authoritative professional (oncologist, palliative care specialist, pain specialist, and others).

For this special issue, the editorial office received forty-six papers and after rigorous peer review process seventeen papers were accepted for publication. The published articles deal with the physiological as well as molecular and biochemical efficacy of medicinal plants and natural active compounds in cancer and tumorigenesis treatment and prevention in vitro and in vivo. Out of the seventeen accepted manuscripts, two articles are very informative high-class review articles. Seven papers used in vivo rat model for their studies; four out of them used clinical trials. In nine publications, the authors used in vitro model utilizing colorectal, hepatoma, squamous carcinoma, leiomyoma, prostate, osteosarcoma, gastric, thyroid, melanoma, and breast cancer cell lines and primary cell culture.

## References

[1] Wang J., Yang D. L., Chen Z. Z., Gou B. (2016). Associations of body mass index with cancer incidence among populations, genders, and menopausal status: a systematic review and meta-analysis. *Cancer Epidemiology*.

[2] Zaid H., Silbermann M., Ben-Arye E., Saad B. (2012). Greco-Arab and Islamic herbal-derived anticancer modalities: from tradition to molecular mechanisms. *Evidence-Based Complementary and Alternative Medicine*.

[3] Daragmeh J., Barriah W., Saad B., Zaid (2016). Targeting the PI3K pathway in tumorigenesis: an in situ comprehensive study. *Oncology Letters*.

